# *Ovostatin 2* knockdown significantly inhibits the growth, migration, and tumorigenicity of cutaneous malignant melanoma cells

**DOI:** 10.1371/journal.pone.0195610

**Published:** 2018-04-23

**Authors:** Ying-Xue Huang, Hao Song, Yue Tao, Xue-Bao Shao, Xue-Si Zeng, Xiu-Lian Xu, Jin-Liang Qi, Jian-Fang Sun

**Affiliations:** 1 Institute of Dermatology, Chinese Academy of Medical Sciences, Nanjing, P. R. China; 2 Department of Dermatology, Xiangya Hospital, Central South University, Changsha, P. R. China; 3 Drum Tower Hospital, Medical School of Nanjing University, Nanjing, P. R. China; 4 State Key Laboratory of Pharmaceutical Biotechnology, School of Life Sciences, Nanjing University, Nanjing, P. R. China; Duke University School of Medicine, UNITED STATES

## Abstract

**Background:**

We previously identified ovostatin 2 (*OVOS2*) as a new candidate gene for cutaneous malignant melanoma (CMM) in a Chinese population. In this study, we aimed to investigate the exact role of *OVOS2* in cell proliferation, invasion, and tumorigenesis of melanoma A375 cells.

**Methods:**

The downregulation of *OVOS2* expression was performed using lentiviral vectors with specific shRNA. The effects of *OVOS2* expression on cell proliferation, cell cycle, cell migration, cell invasion, and potential of tumorigenesis were further investigated.

**Results:**

The downregulation of *OVOS2* significantly suppressed the proliferation of A375 cells and led to a G2/M phase block. The transwell cell migration assay showed that the reduced expression of *OVOS2* also significantly inhibited the transmigration of A375 cells. The western blot results showed downregulated expression of p-FAK, p-AKT, and p-ERK. This was accompanied by the upregulated epithelial phenotypes E-cadherin and β-catenin, and downregulated expression of mesenchymal phenotype N-cadherin after *OVOS2* knockdown. The transplantation tumor experiment in BALB/C nude mouse showed that after an observation period of 32 days, the growth speed and weight of the transplanted tumors were significantly suppressed in the BALB/c nude mice subcutaneously injected with *OVOS2* knocked-down A375 cells.

**Conclusion:**

The inhibition of *OVOS2* had significant suppressive effects on the proliferation, motility, and migration capabilities of A375 cells, suggesting a crucial promotive role of *OVOS2* in the pathogenesis and progression of CMM. The involved mechanisms are at least partly associated with the overactivation of FAK/MAPK/ERK and FAK/PI3K/AKT signals.

## Introduction

Cutaneous malignant melanoma (CMM) is a highly aggressive malignancy arising from the melanocytes, and it is the fifth most frequently diagnosed cancer in human beings[1]. The disease progresses rapidly with a tendency for early metastasis and very few traditional therapeutic options are available for patients in the metastatic stage[2]. In the past few years, numerous molecular markers for melanoma were developed by various chip technologies, accompanied by great advances in molecular therapy[3–5]. However, given that the disease is largely incurable and the underlying mechanisms remain unclear, efforts are still needed to develop novel diagnostic markers and key therapeutic targets for CMM.

In our previous study, a new candidate gene ovostatin2 (*OVOS2*) was identified to be dramatically up-regulated in acral melanomas. The gene was also proved to be correlated with the aggressiveness of CMM[6]. However, the exact roles of *OVOS2* in cell growth, invasion, and tumorigenesis of melanoma remain unclear. OVOS2 is a serine protease inhibitor belonging to the alpha-2-macroglobulin (α2-M) family that can strongly inhibit the activity of proteinases. The activated α2-M could bind to GRP78 present on the surface of cancer cells, and promote cellular proliferation by activating signaling cascades, including MAPK and AKT-dependent signaling[7–12]. It has been demonstrated that GRP78 is over-expressed in cancer cells and is related to the progression of melanoma[13, 14]. In this study, we aimed to examine the crucial roles of *OVOS2* in tumor initiation and progression and to explore whether *OVOS2* has similar effects on MAPK/AKT pathway as α2-M does by performing lentiviral-mediated shRNA interference with *OVOS2* expression. We designed a series of studies to examine the effect of OVOS2 on the malignant phenotype of A375 cells, cell proliferation, cell cycle, cell migration, and invasion. Moreover, we examined the activation of MAPK and AKT-dependent signaling, and the tumorigenic potential of melanoma A375 cells. Based on these experiments, we hope to provide new insights into molecular mechanisms of *OVOS2* in tumor progression.

## Materials and methods

The protocol for the research project has been approved by the Ethics Committee of the Institution of Dermatology (Hospital), Chinese Academy of Medical Sciences and Peking Union Medical College (Permit Number: 200911), and it conforms to the provisions of the Declaration of Helsinki in 1995.

### Cell lines and culture conditions

The melanoma cell lines SK-mel-1 (ATCC® HTB-67™) and A375 (ATCC® CRL-1619™) were obtained from the American Type Culture Collection and preserved in our lab. MV3 and M14 were donated by the lab of department of dermatology, the first affiliated hospital of nanjing medical university and long-term preserved in our lab. Cells were cultured in Dulbecco’s Modified Eagle’s Medium (DMEM; Gibco, CA, USA) supplemented with 10% fetal calf serum. Normal melanocytes were cultured in 254 Medium (Gibco, CA, USA) supplemented with 10% human melanocyte growth supplement.

### Transfection of lentiviral vectors with shRNA for OVOS2

To silence *OVOS2* expression in melanoma cells, we constructed four shRNA–*OVOS2* lentiviral vectors based on the shRNAi vector pGMLV (pGMLV-GFP–vshRNA–*OVOS2*, Genomeditech, Shanghai, China). The four shRNA-targeting sequences for *OVOS2* were as follows: (1) 5′-GCAGTATGTTCTGCTGATTCC-3′; (2) 5′-GCTTGGAACTGTAAACTTTGA-3′; (3) 5′-GGTGAACAAGTTGTTGCAACT-3′; and (4) 5′-GGAACGTCCTTCTACTGAAAT-3′. The recombinant pGMLV–GFP–vshRNA–*OVOS2s* were identified by PCR and DNA sequencing. Lentivirus packaging was conducted in 293T cells, followed by transfection with the four shRNA–*OVOS2*s into A375 cells. A mock lentiviral vector was used as a negative control. The optimum shRNA fragment against *OVOS2* was determined by real-time PCR. The interference of this selected shRNA on *OVOS2* was verified by immunocytochemistry.

### Cellular experiments

#### RNA extraction and real-time PCR analysis

The total RNA extraction and real-timem PCR were performed as described previously[6]. β-actin gene, which has been proved to be a housekeeping gene in human tissues, was used as an internal control to normalize the variation in the amount of cDNA template. Normal melanocyte was used as a control to calculate the relative mRNA levels of the melanoma cell lines. The untreated cell was used as a controlfor cells transfected by lentiviral vectors with shRNA for *OVOS2*. The fold change of *OVOS2* mRNA expression in the tested sample was calculated using the following formulas: *OVOS2*ΔCt ¼ = (Avg. *OVOS2* _Ct-Avg. β-actin_Ct) and *OVOS2*ΔΔCt = (*OVOS*2ΔCt_tested sample-*OVOS*2ΔCt_control). Then we got a normalized *OVOS2* amount relative to the control, which is shown as 2^-ΔΔCt^. The mean from three independent experiments was calculated.

#### Western blot analysis

Cellular protein was extracted from cultured cells by using a total protein extraction kit (KeyGen Biotech, China) and quantified by the Bradford protein assay kit (KeyGen Biotech). The western blotting procedure was the same as described previously[6]. β-actin or GADPH was used as an internal control to confirm equal loading of the samples. A rabbit polyclonal anti-OVOS2 antibody against synthetic peptide conjugated to KLH, corresponding to a region within N terminal amino acids 57–87 of Human OVOS2, was used as the primary antibody at a diluted ratio of 1:500 (Abcam, Cambridge, UK; RRID:AB_10861079). The other primary antibodies were the diluted solution of antibodies that recognize AKT (1:500), p-AKT (1:500), ERK (1:3000), p-ERK (1:800), FAK (1:1000), p-FAK (1:500), cyclin A (1:500), cyclin B (1:1000), cyclin D1 (1:1000), CDK2 (1:800), matrix metalloproteinase-2 (MMP-2; 1:500), N-cadherin (1:1000), E-cadherin (1:1000), and β-catenin (1:1000), and they were obtained from Affinity (OH, USA). The HRP-conjugated anti-rabbit IgG secondary antibody was obtained from KeyGen BiotechCo. Ltd.

#### Immunocytochemistry

The cells were cultured on coverslips. After cell attachment and formation of a confluent monolayer, the cells were immersion fixed in 95% alcohol for 30 min. The coverslips were washed three times (5 min each) with phosphate-buffered saline (PBS) and then incubated with 1 mM Triton-100 for 20 min to enhance the permeability of the cell membrane. After three washes in PBS (5 min each), the coverslips were incubated with the rabbit polyclonal antibody against OVOS2 (1:100) at 25°Cfor 1 h. Afterward, the coverslips were washed three times (5 min each) in PBS and then incubated for 15 min at room temperature with HRP-labeled antibodies. After three washes (5 min each) with PBS, the coverslips were stained in a peroxidase detection system. The antibody binding was visualized using diaminobenzidine as the chromogen, and counterstaining was performed with hematoxylin. Immunostaining with PBS instead of primary antibody served as the negative control.

#### MTT cell viability assay

The cells were seeded in triplicate into a 96-well plate (5,000 per 200 μL/well) and incubated for 24, 48, 72, and 96 h. Aliquots of 20 μL of 0.5 mg/mL 3-(4,5-dimethylthiazol-2-yl)-2,5-diphenyltetrazolium-bromide (MTT) (Sigma–Aldrich, St. Louis, MO, USA) solution were added at the indicated time points. Approximately 4 h later, the medium was replaced with 150 μL dimethyl sulfoxide (DMSO) and vortexed for 10 min. The absorbance (A) at 490 nm was measured to determine the number of viable cells in each well. The viability of cells in the different groups was compared at specified time points.

#### *In vitro* assay for cell growth

Cells were counted, diluted, and seeded in triplicate at 50000 cells per D-35mm culture dish in 1 ml DMEM-10% serum. Cell growth was measured by counting cell numbers.

#### Colony formation assay

Approximately 1000 mock or transfected cells were plated by serial dilution in triplicate in 6 cm culture dishes without any substrate. After 14 days, the cells were stained with 0.1% crystal violet, and the visible colonies were manually counted. The experiment was performed in triplicate.

#### Apoptosis detection

Staining with annexin V and 7-aminoactinomycin D (**7-**AAD) were performed to evaluate chromatin condensation according to the manufacturer’s instructions (BioLegend, CA, USA). Briefly, the cells were washed in ice-cold PBS and resuspended in the binding buffer at a density of 1×10^6^ cells/mL. Then, the cells were stained with 5 μLannexin V-FITC and 10 μL 7-AAD for 15 min. Flow cytometric analysis was performed on a FACSCalibur (Becton Dickinson, NJ, USA), and the fraction of cells in each quadrant was calculated using the CellQuest Pro software (Becton Dickinson).

#### Caspase-3 activity analysis

The activity of caspase-3 was measured using the colorimetric Caspase-3 Assay kit (Keygen, Nanjing, China) according to the manufacturer’s instructions. Briefly, equal amounts of cell extracts prepared from cells were incubated with the Caspase-3 substrate in the assay buffer for 4 h at 37°C. Absorbance was measured at 400 nm using a Multiscan Ascent (Thermo, MA, USA). Untreated A375 cell was used as a reference for results analysis.

#### Cell cycle detection

The harvested cells were fixed in 75% ethanol overnight at -20°C. Then, they were washed with PBS, digested by DNase-free RNase A, and stained with 50 mg/mL propidium iodide (PI; Beckman Coulter, CA, USA) in PBS at 4°C for 30 min. Flow cytometric analysis was performed on a FACScan system (Becton Dickinson), and the data were analyzed by using the ModFit software (Verity Software House, ME, USA).

#### Cell-matrix adhesion assay

For adhesion assay, Matrigel (BD Biosciences, CA, USA) was used as the adhesion substrate, the major component of which is laminin, followed by collagen IV, heparan sulfate proteoglycans, entactin/nidogen. It also contains TGF-beta, epidermal growth factor, insulin-like growth factor, fibroblast growth factor and tissue plasminogen activator. It is effective for the attachment of anchorage dependent cells. The cells were pretreated with serum-free DMEM for 24 h and then detached from the culture dishes by trypsin. Subsequently, 2 × 10^4^ cells were seeded in a 96-well microplate coated with 0.5 mg/mL of Matrigel and left to adhere for 1 h in a humidified atmosphere at 37°C and 5% CO_2_. The cells were then washed 3 times with sterile PBS to remove any detached cells. The adhered cells were incubated at 37°C with the complete medium, and MTT assay was performed as described above. The experiment was performed in triplicate.

#### Cell migration assays

A monolayer wound-healing assay was used to evaluate the cell migration ability[15]. The cells were grown to 80–90% confluence in 6-well plates. A wound was made by dragging a plastic pipette tip across the cell surface. The remaining cells were washed three times to remove any cell debris and incubated at 37°C with serum-free DMEM. At 6, 12, and 24 h, six different cleared zones per well of migrating cells at the wound front were photographed and compared. The cell migration distance was determined by measuring the width of the wound and subtracting half this value from the initial half-width value of the wound. Each experiment was performed in triplicate and three separate experiments were performed.

#### Cell transmigration and invasion assays

The transmigration and invasion assays were conducted in 24-well transwell chambers (8μm pore size) using an uncoated membrane and Matrigel-coated membrane (BD Biosciences), respectively. The cells (1×10^5^ cells) were then suspended in 200 μL of serum-free DMEM and added to the upper chamber, while 500 μL of complete DMEM was placed in the lower chamber to serve as a chemoattractant stimulus. After 24 or 48 h of incubation, the nonmigratory or noninvasive cells remaining in the upper chamber were removed by cotton-tipped swabs. The transmigrated or invaded cells that had adhered to the bottom surface of the chamber membranes were stained with crystal violet. The cells in five randomly selected microscopic fields were counted and photographed. All experiments were performed three times in triplicate for each sample.

#### Animal experiments

All animal experiments were ethically acceptable as they were performed in full compliance with the national guidelines for animal usage in research and with the approval of the Animal Care and Use Committee, Institute of Dermatology (Hospital), Chinese Academy of Medical Sciences and Peking Union Medical College (Permit Number: 200911).

Female BALB/c nude mice, obtained from the laboratory animal center of Academy of Military Medical Sciences (Beijing, China), were kept in a special pathogen-free facility, which is accredited for animal care by the Chinese Association for Accreditation of Laboratory Animal Care.

For the tumor formation assay, 18 five- to six-week-old BALB/c nude mice were divided randomly into three groups, then A375/*OVOS2*–shRNA cells, A375/NC–shRNA cells, or untreated A375 cells were subcutaneously injected at a dose of 2 × 10^7^ cells per animal for each group. Each animal received one injection in the armpit. The primary tumor size was determined every other day as 3D measurements (mm) using a caliper. Only measurable tumors were used to calculate the mean tumor volume for each tumor cell clone at each time point. The animals were euthanized by carbon dioxide inhaling 32 days after the injection, when the largest tumors reached approximately 15 mm in diameter. All the transplanted tumors were fixed in 10% formalin and embedded in paraffin. The invasive or metastatic patterns were analyzed using hematoxylin-eosin (H&E) staining. The mitotic rate was evaluated in five randomly selected high-power fields under a light microscope.

Immunohistochemical staining was performed in the tissues of the transplanted tumors as described previously[6], and the procedure was similar to that of immunocytochemistry mentioned above. Briefly, the tissue sections were dewaxed and rehydrated. Antigen-epitope retrieval was performed by using 10 mmol/L citric acid buffer solution (pH 6.0), followed by overnight incubation at 4°C with the primary antibodies, and then incubated with HRP-labeled secondary antibody for 15 min at room temperature after three washes (5 min each) with PBS. The peroxidase detection system was used to stain the slices and hematoxylin was used as a counterstain. The antibodies that recognize Ki-67, Bcl-2, Bax, E-cadherin, β-catenin and the HRP-conjugated IgG secondary antibody were ready-to-use and obtained from Maixin Biotech (Fuzhou, China). The results were quantified blindly and independently by two independent observers. The disagreements among the observers were discussed under a multi-scope microscope for a final consensus. The mean percentage of the positive cells was determined in at least four random areas at a magnification of 400 ×. The staining intensity was scored as negative (0), weak (1), moderate (2), and intense (3). The scores for staining intensity and the percentage of positive cells were multiplied to obtain a final score for each case. For Ki-67, the Ki-67 labeling index (KI) was calculated as the percentage of Ki-67-positive nuclei per 1000 tumor-cell nuclei[16].

### Statistical analysis

Statistical analyses were performed using the SPSS 17.0 software (IBM, IL, USA). One-way ANOVA, LSD multiple comparison, and Kruskal–Wallis test were used for comparison between the groups. All tests were two-tailed, and *P* < 0.05 was considered as statistically significant.

## Results

### Expression of *OVOS2* mRNA and protein in melanoma cell lines

**Fig 1:**
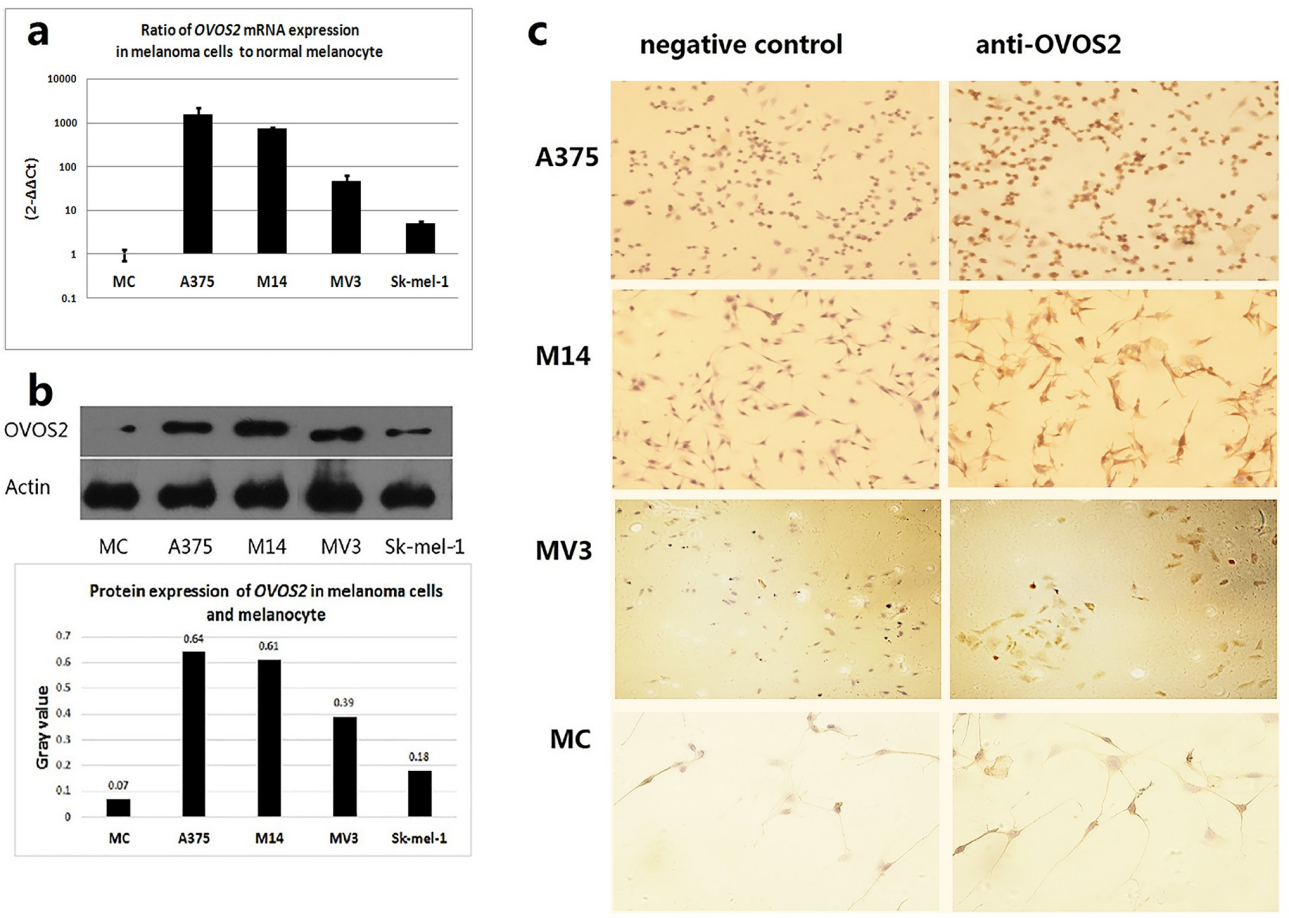
OVOS2 expression in different melanoma cell lines. (**a**) *OVOS2* mRNA level in the four melanoma cell lines: A375, M14, MV3, and Sk-mel-1. The expression levels are shown relative to the primary cultured melanocytes. (**b**) OVOS2 protein expression analyzed using western blotting, with β-actin as the loading control. (**c**) OVOS2 protein expression measured by immunocytochemical analysis using polyclonal anti-OVOS2 antibody (magnification of A375 and MV3: 100×; M14 and MC: 200×). Sk-mel-1 was not tested for using immunocytochemistry because of being suspension-cultured.

As shown by real-time PCR and western blot analysis (Fig 1A and 1B), significantly upregulated levels of *OVOS2* mRNA and protein were detected in all four melanoma cell lines compared with those in the primary melanocytes. Moreover, good concordance between the mRNA and protein levels was observed in all the cell lines. Of the four kinds of melanoma cells, A375 presented the highest expression level of *OVOS2*. Further immunocytochemical examination confirmed that the cytoplasm of the melanoma cell lines A375, MV3, and M14, but not of the primary cultured melanocytes, was stained with anti-OVOS2 antibody (Fig 1C).

### Knockdown of OVOS2 expression by lentiviral vector with shRNA in A375 melanoma cells

**Fig 2:**
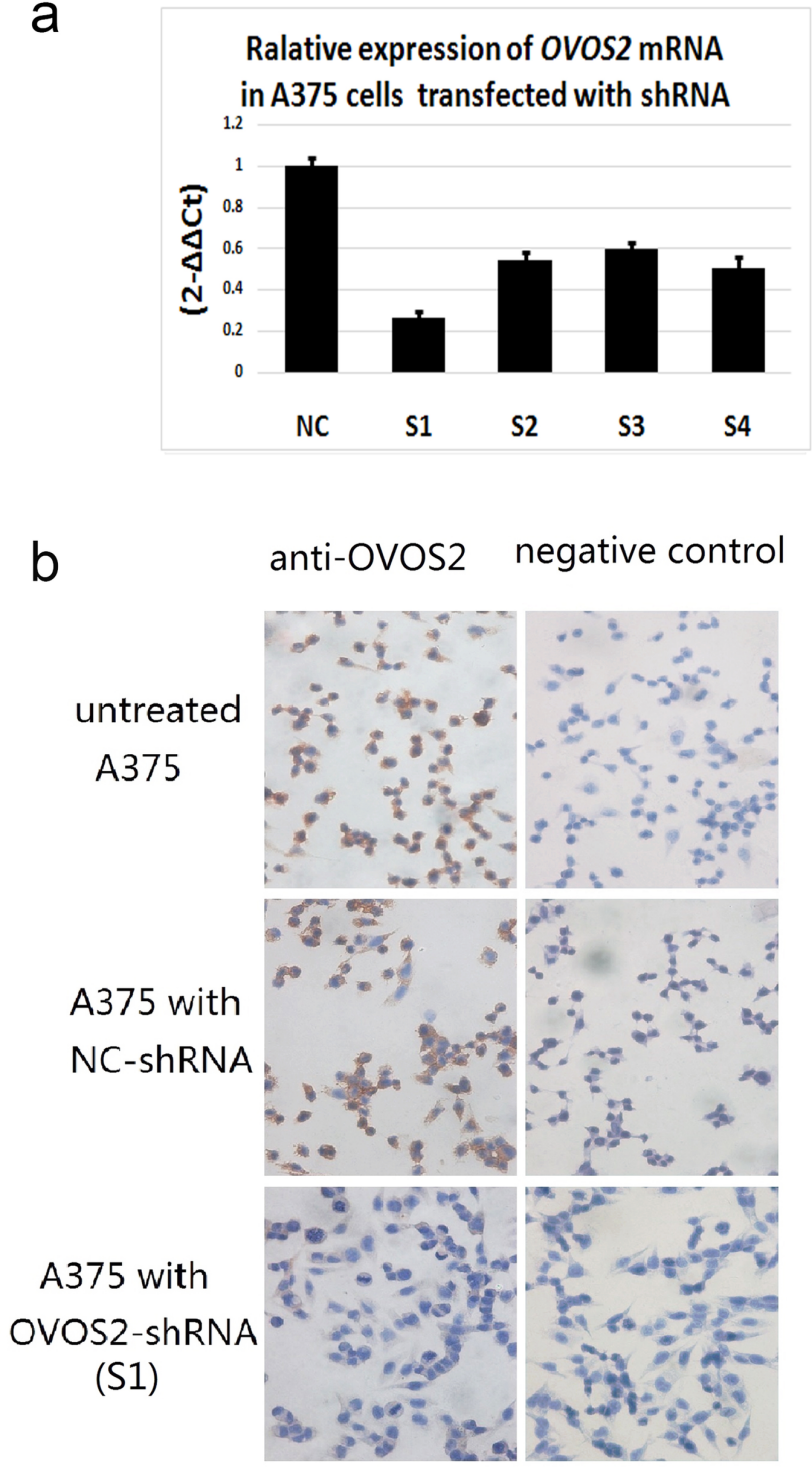
OVOS2 expression in A375 cells transfected with or without *OVOS2-*shRNA. (**a**) A decrease of *OVOS2* mRNA was observed after transfection with four *OVOS2*-shRNAs (S1, S2, S3, and S4) in A375. The expression levels are shown relative to A375 cells transfected with the negative control-shRNA (NC). Then the stable transfectants of *OVOS2-*shRNA1 (hereafter referred to as *OVOS2*-shRNA) were chosen for further study. (**b**) OVOS2 protein expression measured by immunocytochemical analysis in untreated A375 cells (100×), A375 cells transfected with NC-shRNA (100×), and A375 cells transfected with *OVOS2*-shRNA (S1) (200×).

Four lentiviral vectors with shRNA for *OVOS2* were constructed successfully. Melanoma A375 cells, which presented the highest expression level of *OVOS2*, were selected as the target cells. Real-time-PCR showed that the expression of *OVOS2* could be significantly inhibited by all four shRNA fragments (Fig 2A), of which *OVOS2*–shRNA1 exhibited the most efficient inhibition of *OVOS2*. Immunocytochemistry confirmed that *OVOS2* expression was noticeably inhibited in *OVOS2*–shRNA1-transfected A375 cells (Fig 2B). Therefore, the corresponding stable transfectants of *OVOS2*-shRNA1 (hereafter referred to as *OVOS2*-shRNA) were chosen for further study.

### OVOS2 was involved in the cell proliferation and cell cycle process of A375, but it weakly affected the cell apoptosis rate

**Fig 3:**
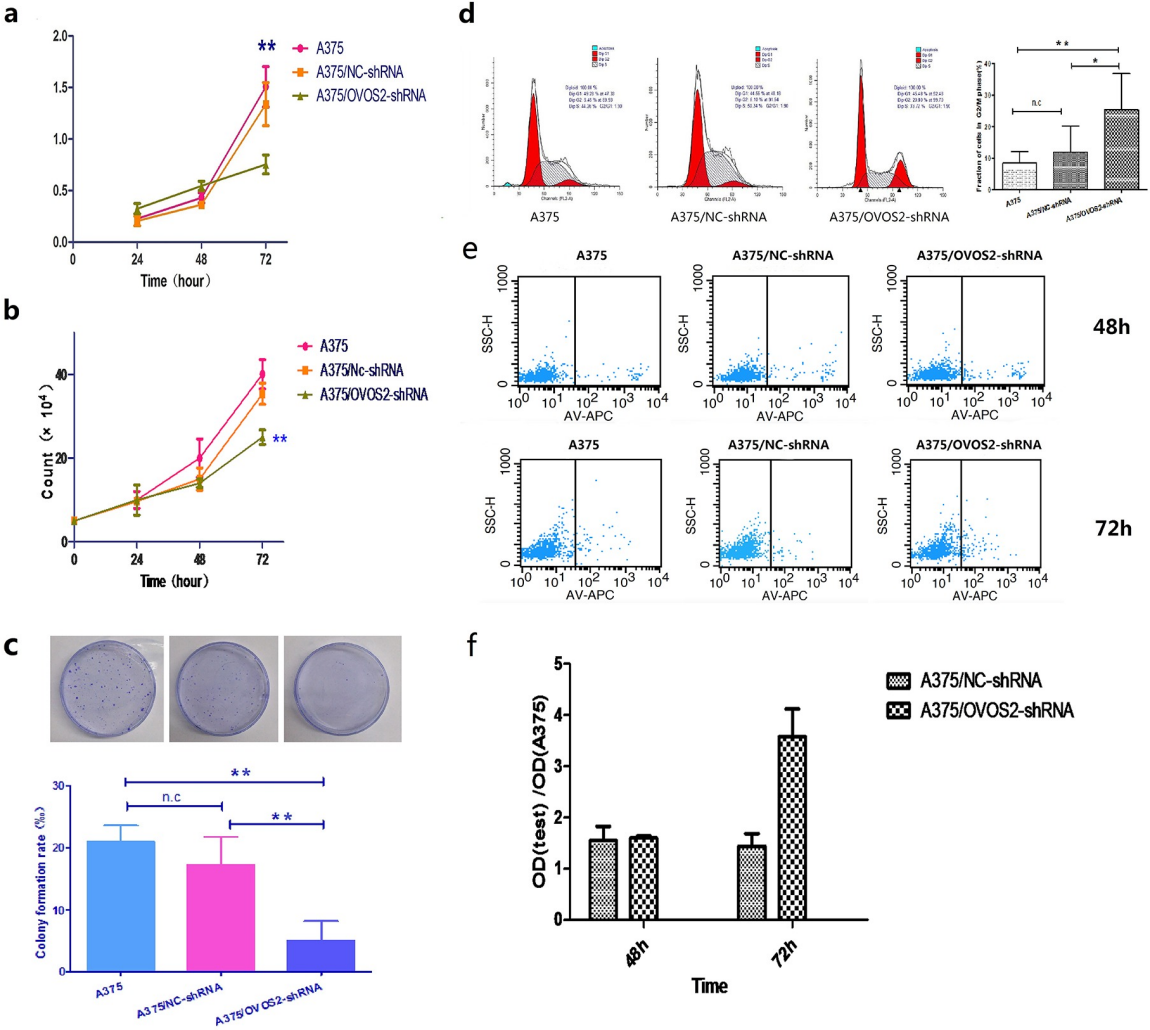
Effect of *OVOS2* downregulation on cell proliferation, cell cycle, and apoptosis of A375. **There are representative results from three independent experiments.** (**a**) The cell proliferation rate evaluated by MTT assay was significantly inhibited after 72 h of incubation in A375 cells transfected with the *OVOS2*-shRNAs, while no significant difference was observed at 24 or 48 h (**P < 0.01, ANOVA, LSD multiple comparison). (**b**) Results of cell number counting test. The proliferative rate at 72 h significantly decreased in A375/*OVOS2*–shRNA compared with that in A375/NC–shRNA and untreated A375 cell groups (**P < 0.01, rANOVA, LSD multiple comparison). (**c**) The colony formation assay showed the colony forming activity during the 14 d culture of A375 cells, A375/NC-shRNA and A375/*OVOS2*-shRNA (**P < 0.01, ANOVA, LSD multiple comparison). (**d**) Flow cytometric analysis showed significant accumulation of G2/M phase fraction at 72 h in A375 transfected with *OVOS2*-shRNA (**P < 0.01, *P < 0.05, ANOVA, LSD multiple comparison). (**e**) Flow cytometer analysis by staining with AnnexinV and 7-AAD. In the scatter plots, the left quadrant represents viable cells, and the right quadrant represents apoptotic cells. The results showed an unaffected apoptosis rate at 48 h and a non-significant increased apoptosis rate at 72 h in A375/OVOS2-shRNA, compared with A375 cell and A375/NC-shRNA (*P* > 0.05, ANOVA). (**f**) Results of Caspase-3 activity analysis. Downregulation of *OVOS2* did not significantly affect the percentage of apoptotic cells after 48 (*P*>0.05, ANOVA) or 72 h culture (*P* = 0.05, ANOVA).

The MTT assay showed that the proliferative rate at 72 h significantly decreased in A375/*OVOS2*–shRNA compared with that in the A375/NC–shRNA (Fig 3A). The cell number counting gave results similar to those of the MTT test (Fig 3B). The colony formation assay showed that the colony forming activity of A375/*OVOS2*–shRNA was significantly inhibited (Fig 3C). The above results of these *in vitro* studies demonstrated the proliferation potential of A375 cells was significantly inhibited after *OVOS2* silencing. Regarding the cell cycle, flow cytometry revealed a G2/M phase block in A375/*OVOS2*–shRNA at 72 h (Fig 3D). However, the downregulation of *OVOS2* did not significantly affect the percentage of apoptotic cells at 48((6.253+2.508)% *vs* (8.047+2.879)% and (8.07+1.202)%, *P* = 0.39) and 72 h((12.6±4.5)% *vs* (6.5±1.7)% and (6.7±2.8)%, *P* = 0.082) as shown by the flow cytometric analysis (Fig 3E). Similar results were obtained by caspase-3 activity analysis (Fig 3F).

### OVOS2 was involved in cell motility and migration of A375, but it weakly affected the Matrigel invasion

**Fig 4:**
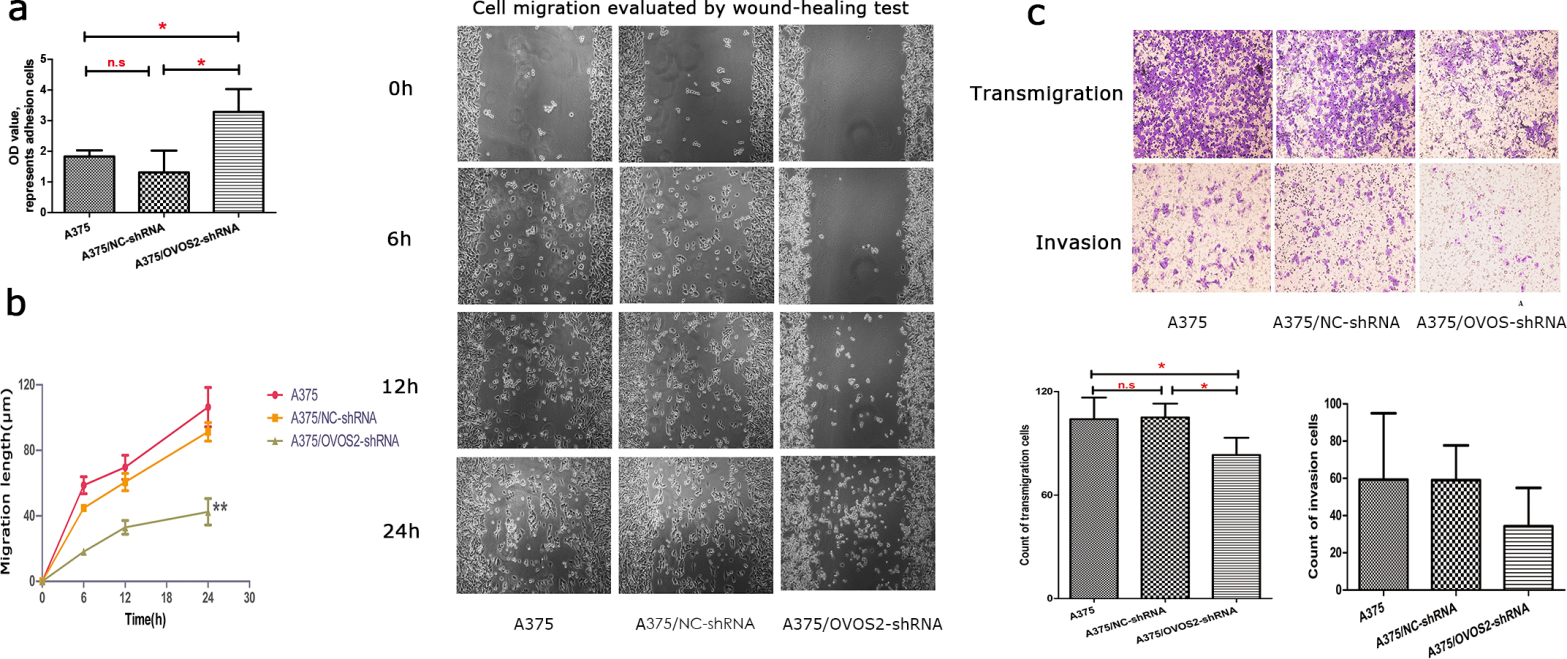
Effect of *OVOS2* downregulation on the cell invasive behavior of A375. (**a**) The enhanced cell-matrix adhesion rate of A375 cells transfected with *OVOS2*-shRNA was shown (*P < 0.05, *P* = 0.007 and *P* = 0.026, compared with A375/NC-shRNA and untreated A375 cell groups, respectively; ANOVA, LSD multiple comparison). The average was calculated from three independent experiments and presented with standard deviation. (**b**) The wound healing assay showed the inhibition of cell motility in A375 cells following *OVOS2* downregulation with shRNA (**P<0.01, repeated measure of ANOVA). Representative results from three independent experiments were shown (100×). (**c**) The inhibition of the transmigration ability during 24 h culture of A375 cells transfected with *OVOS2*-shRNA was shown using the Transwell migration study (*P < 0.05, *P* = 0.016 and *P* = 0.02, compared with A375/NC-shRNA and untreated A375 cell groups, respectively; ANOVA, LSD multiple comparison); and a nonsignificant decrease of invasion ability during 48 h culture of A375 cells transfected with *OVOS2*-shRNA was shown by the Matrigel invasion assay (*P* = 0.453, ANOVA). Representative results from three independent experiments (200 ×).

The adhesion assay revealed that the cell-matrix adhesion was significantly stronger in A375/*OVOS2*–shRNA (Fig 4A). The wound healing study showed that the migration distance at 6h, 12h and 24h of A375 cell, A375/NC-shRNA and A375/OVOS2-shRNA were (58.68±22.10, 69.62±31.38, 106.30±50.87), (44.76±7.68, 60.59±18.36, 91.27±19.45) and (18.16±9.33, 32.95±17.75, 42.46±34.42), indicating the wound closure of A375/*OVOS2*–shRNA cells was markedly delayed at the indicated time points compared with that of the A375/NC–shRNA and untreated A375 cells (both *P*<0.001 at 6h, P = 0.003 and *P*<0.001 at 12h, *P* = 0.002 and *P*<0.001 at 24h, respectively = (Fig 4B), and similar results were obtained by wound healing assay in complete medium (a supporting information file shows this in more detail [see S1 Fig]), further supporting the significant suppression of cell motility ability by OVOS2 knocked-down. Transwell migration study showed that the number of transmigrated A375/*OVOS2*–shRNA cells decreased significantly (*P* = 0.016 and *P* = 0.02, compared with A375/NC-shRNA and untreated A375 cell groups, respectively) (Fig 4C). In the Matrigel invasion study, a decrease in cell invasion was observed, although the differences were not significant (*P =* 0.453; Fig 4C).

### *OVOS2* silencing affected the expression of cell cycle-related proteins in A375 cells

**Fig 5:**
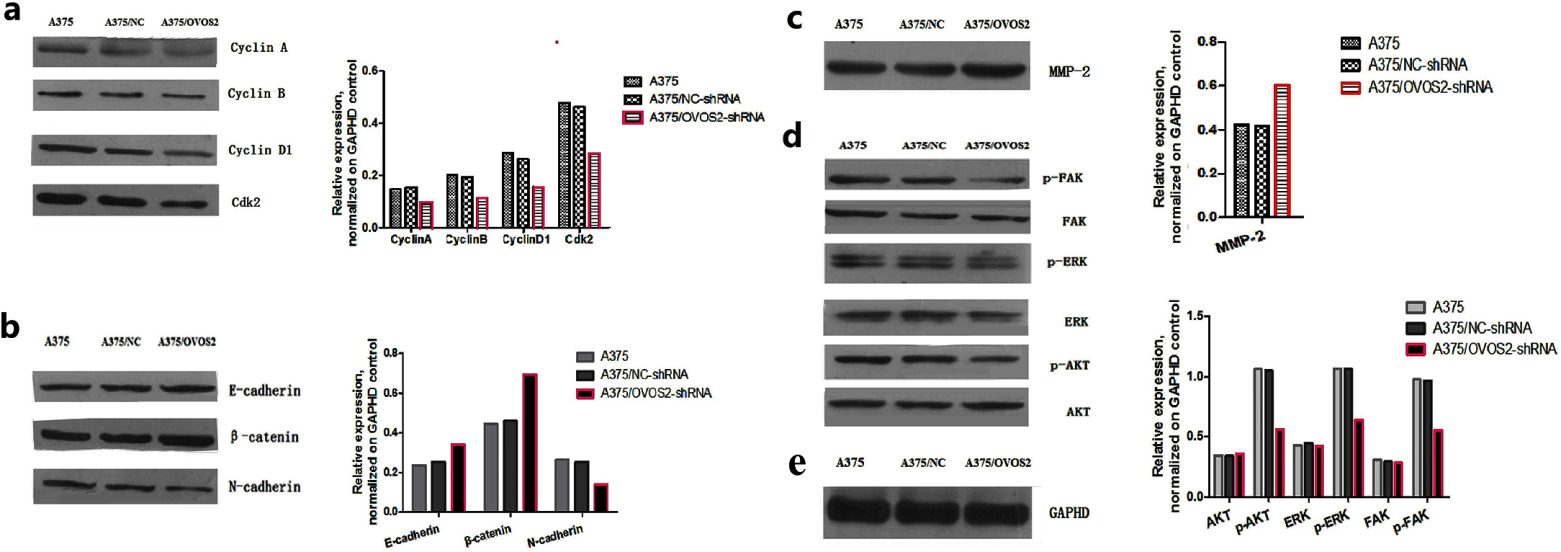
Western blot results. (**a**) The levels of cyclin A, cyclin B, cyclin D1, and CDK2 were reduced in A375 cells transfected with *OVOS2*-shRNA; (**b**) The downregulated expression of N-cadherin accompanied with the upregulated expression of E-cadherin and β-catenin were observed in A375 cells transfected with *OVOS2*-shRNA; (**c**) The expression of p-FAK, p-AKT, and p-ERK were reduced in A375 cells transfected with *OVOS2*-shRNA; (**d**) The increased production of MMP-2 was observed in A375 transfected with *OVOS2*-shRNA; (**e**) GAPDH was used as the reference.

As shown by western blot analysis (Fig 5A), the expression levels of the regulators of cell cycle including cyclin A, cyclin B, cyclin D1, and CDK2 were reduced after *OVOS2* was knocked down in A375 cells.

### OVOS2 affected the expression of proteins related to invasiveness in A375 cells

Western blot revealed a downregulated expression of the mesenchymal phenotype N-cadherin, accompanied by the upregulated expression of the epithelial phenotypes E-cadherin and β-catenin (Fig 5B), and increased production of proteinase MMP-2 after *OVOS2* silencing in A375 cells (Fig 5C).

### *OVOS2* silencing suppressed the phosphorylation of AKT, ERK, and FAK in A375 cells

*OVOS2* silencing reduced the expression of key cancer related signal markers p-FAK, p-AKT, and p-ERK, without affecting the expression of AKT, ERK, and FAK in A375 cells, indicating a possible role of OVOS2 in activating these pathways. (Fig 5D).

### OVOS2 expression affected the tumorigenicity of A375 cells in vivo

**Fig 6:**
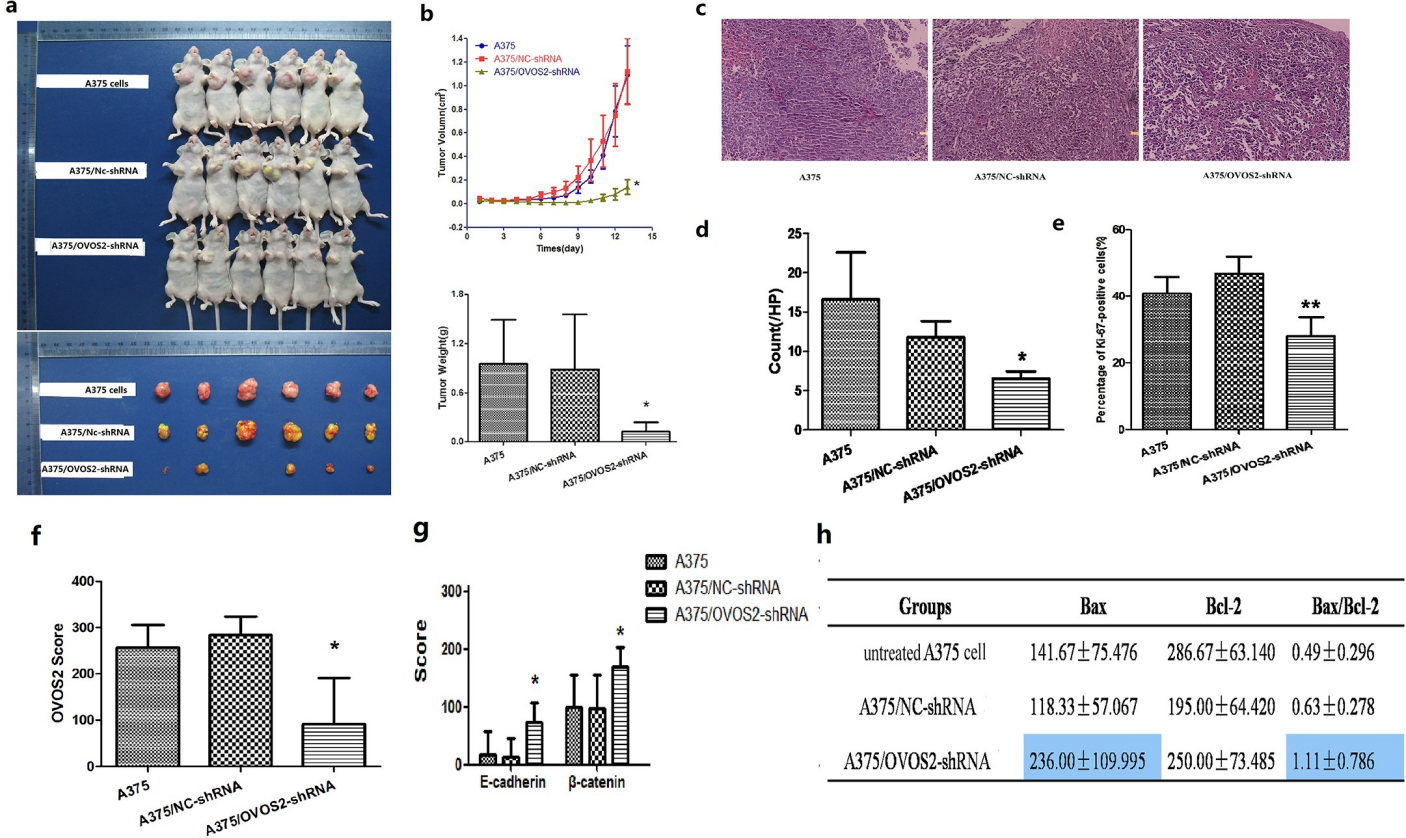
BALB/C nude mouse subcutaneous transplantation tumor experiment and immunohistochemistry of the transplanted tumors. (**a**) All the mice from the three groups developed tumors. *OVOS2* downregulation significantly decreased tumor growth and one tumor spontaneously regressed in the A375/*OVOS2*-shRNA group. (**b**) The tumor growth was significantly inhibited in the A375/*OVOS2*-shRNA group during the observation period (*P < 0.05, *P* = 0.011 and *P* = 0.03, compared with A375/NC-shRNA and untreated A375 cell groups, respectively; repeated measure of ANOVA); the weight of tumors in the A375/*OVOS2*-shRNA group was also significantly reduced (*P < 0.05, *P* = 0.019 and *P* = 0.012, compared with A375/NC-shRNA and untreated A375 cell groups, respectively; ANOVA, LSD multiple comparison). The results are shown as means ± SD. (**c**) H&E staining of the three groups (100×). The histopathologic characteristics of the xenograft tumors among the three groups were similar. (**d**) Number of abnormal mitoses of transplanted tumors in every high power field. The number of abnormal mitoses was much less in the A375/*OVOS2*–shRNA tumors compared with those in the A375 cell and A375/NC–shRNA tumors (*P < 0.05, *P* = 0.002 and *P* = 0.023, compared with A375/NC-shRNA and untreated A375 cell groups, respectively; ANOVA, LSD multiple comparison). (**e**) The downregulation of *OVOS2* in A375 cells dramatically decreased the percentage of Ki-67 positive cells as shown by immunohistochemistry study (**P < 0.01, ANOVA, LSD multiple comparison). (**f**) Immunohistochemistry staining of OVOS2 expression in transplanted tumors. OVOS2 was expressed in tumors induced by A375 and A375/NC–shRNA cells, but it was absent in one of the A375/*OVOS2*–shRNA cell-induced tumors, and the expression level in the group of A375/*OVOS2*–shRNA was significantly decreased (*P < 0.05, *P* = 0.009 and *P* = 0.033, compared with A375/NC-shRNA and untreated A375 cell groups, respectively; Kruskal–Wallis test). (**g**) The expression of E-cadherin and β-catenin in the xenograft tumors showed that both E-cadherin and β-catenin were significantly upregulated in the A375/*OVOS2*-shRNA tumor (*P < 0.05, *P* = 0.015 and *P* = 0.02 for E-cadherin, *P* = 0.033 and *P* = 0.04 for β-catenin, compared with A375/NC-shRNA and untreated A375 cell groups, respectively; ANOVA, LSD multiple comparison). (**h**) Immunohistochemistry staining for apoptosis indexes of Bax and Bcl-2in transplanted tumors. The ratio of Bax to Bcl-2 was higher in the A375/*OVOS2*–shRNA group than that in the A375 and A375/NC–shRNA groups; but not reached statistical significant difference (*P*>0.05, Kruskal–Wallis test).

After 5–7 days of a lag phase, all the mice in the three groups developed tumors successively. Although no difference in tumor incidence was observed among the groups (Fig 6A), tumor growth was significantly inhibited in the A375/*OVOS2*–shRNA group during the observation period (*P* = 0.011 and *P* = 0.03, compared with A375/NC-shRNA and untreated A375 cell groups, respectively Fig 6B). The tumor size of the A375/*OVOS2*–shRNA group was only 10% of the parental A375 and A375/NC–shRNA tumors. Notably, spontaneous tumor regression was observed in one mouse in the A375/*OVOS2*–shRNA group. The tumor weight was also significantly reduced, with an inhibition of more than 85% in the A375/*OVOS2*–shRNA group (*P* = 0.019 and *P* = 0.012, compared with A375/NC-shRNA and untreated A375 cell groups, respectively; Fig 6B). No difference in body weights was observed among the three mice groups. The histologic appearances of the transplanted tumors among the three groups were undistinguishable (Fig 6C). The number of abnormal mitoses in all high-power fields was much less in the A375/*OVOS2*–shRNA tumors compared with those in the A375 cell and A375/NC–shRNA tumors (Fig 6D).

### Immunohistochemistry of the transplanted tumors

OVOS2 was expressed in tumors induced by A375 and A375/NC–shRNA cells, but it was absent in one of the A375/*OVOS2*–shRNA cell-induced tumors, and the expression level in the group of A375/*OVOS2*–shRNA was significantly decreased (Fig 6F). Immunoreactivity of the proliferative marker Ki-67 was detected in the nuclei of cancer cells in all the tumors; however, the KI was significantly lower in the A375/*OVOS2*–shRNA group than that in the A375 and A375/NC–shRNA groups (*P* = 0.001 and *P* < 0.001, compared with A375/NC-shRNA and untreated A375 cell groups, respectively; Fig 6E). This finding was in accordance with the abnormal mitosis number. Immunohistochemistry staining for apoptosis indices in Bax and Bcl-2 revealed that the ratio of Bax to Bcl-2 was higher in the A375/*OVOS2*–shRNA group than in the A375 and A375/NC–shRNA groups. However, the differences were insignificant (Fig 6H), which was consistent with the *in vitro* apoptosis analysis results. Semi-quantitative analyses of the expression of epithelial proteins revealed that E-cadherin and β-catenin were both significantly upregulated in the A375/*OVOS2*–shRNA group tumors than in the A375 and A375/NC–shRNA group tumors (*P* = 0.02 and *P* = 0.015 for E-cadherin; *P* = 0.04 and *P* = 0.033 for β-catenin; Fig 6G), which was in accordance with the western blot result.

## Discussion

The two major hallmarks of malignancy are infinite growth and invasiveness[17]. We conducted a series of studies to examine the effect of *OVOS2* on two phenotypes of melanoma A375 cells.

The infinite growth potential of tumor is considered as the result of uncontrolled proliferation, apoptosis defect, and cell cycle dysregulation. Through a series of *in vitro* and *in vivo* tumorigenesis studies combined with the KI index of the transplanted tumors, we found that the proliferation potential of A375 cells was significantly inhibited after *OVOS2* silencing. The results of the cell cycle study revealed a G2/M phase block in *OVOS2* knockdown cells, whereas the percentage of apoptotic cells remained unchanged. The Bax/Bcl-2 ratio in planted tumors, which represents the level of apoptosis, showed no significant difference as well, from which we concluded that OVOS2 involvement in cell proliferation is mainly attributed to cell cycle dysregulation, rather than the apoptosis defect. Cyclin A and B are key proteins in regulating the cell cycle from G2 entry into M-phase. In the present study, the G2/M phase block accompanied by the downregulated expression of cyclin A and B indicated a cell mitotic arrest caused by *OVOS2* silencing[18, 19]. The reduced expression of cyclin D1 and CDK2, which are the G1/S phase-related regulators of the cell cycle, were also observed after *OVOS2* knockdown. Besides its promoting role in cell cycle, CDK2 is also a downstream product of microphthalmia-associated transcription factor (MITF), which is essential for CMM development. Previous data revealed that the inhibition of *CDK2* could significantly reduce the growth of melanoma cells[20, 21]. In our study, the inhibition of CDK2 may partly contribute to the suppression of cell growth due to *OVOS2* silencing. Cyclin D1 is a key regulator not only promoting G1/S transition, but also regulating the process of tumor progression such as cellular migration[22, 23]. The down-regulation of cyclin D1 due to *OVOS2* silencing may influence the biological behavior of A375 cells independent of cell cycle regulation, but the precise mechanism needs to be studied further.

Tumor invasion, which is another hallmark of malignancy, occurs in a series of discrete steps, including loss of cell adhesion to the matrix, degradation of the extracellular matrix (ECM), and the motility and migration of tumor cells. In this study, we observed that the decreased *OVOS2* expression significantly enhanced cell adhesion but suppressed motility and transmigration, indicating a reduced invasiveness of A375 cells *in vitro*. The cadherins are a super-family of adhesion molecules that play important roles in cell adhesion, cell motility, and tumor invasion[24–26]. In this study, we observed a downregulated expression of N-cadherin, accompanied by an upregulated expression of E-cadherin and β-catenin in *OVOS2*-interfered A375 cells. The upregulated expression of E-cadherin and β-catenin were also observed in transplant tumors by immunohistochemistry. E-cadherin and β-catenin were considered as epithelial proteins, while N-cadherin is a mesenchymal cadherin. The switch from epithelial to mesenchymal phenotype in tumor cells was known as epithelial-mesenchymal transition (EMT), which has been observed as a vital process during melanoma development[27, 28]. Loss of E-cadherin in melanoma can be associated with an increase of mesenchymal phenotypes N-cadherin expression. Lots of in vitro studies have shown that down-regulation of membrane expression of epithelial, or E-cadherin, and increase in neural, or N-cadherin, are involved in the ability of a melanoma cell to migrate out of its nascent environment. One of the important early steps in melanoma development includes disruption of E-cadherin-mediated adhesive interaction between melanocytes and keratincoytes, accompanied by increased expression of N-cadherin, which facilitates proliferation and invasion of melanoma cells[29, 30]. The modulation of cadherins by *OVOS2* silencing observed in the present study could partly explain the inhibition of cell motility and migration. In addition, it can suggest the possible role of OVOS2 in the EMT process of melanoma cells; however, further study with the detections of additional markers of EMT, such as Snail, Twist, and Zeb1, would give more evidences.

MMP-2 is a key promotor in the migration and invasion of tumor cells by degradating ECM[31]. Notably, we revealed that *OVOS2* silencing enhanced MMP-2 expression, which indicated an enhanced proteolytic potential however, the invasive cell number was not significantly decreased as expected in the Matrigel invasion assay. Repeated tests of the expression of MMP-2 and Other MMPs should be further conducted. However, the cell lines cultured in artificial environments cannot fully recreate the invasive process of tumors *in vivo*, further invasive and metastatic studies should be performed on metastatic animal models.

Information on the mechanisms underlying the involvement of OVOS2 in tumor is limited. We assumed OVOS2 might interact with some key cancer related signaling pathways to regulate the cell growth and tumorigenesis. In this study, we demonstrated that the interference of *OVOS2* could dramatically inhibit the phosphorylation of ERK, AKT, and FAK, indicating a possible role of OVOS2 in activating these pathways. ERK1/ERK2 is essential for G1/S phase and G2/M phase transition and plays a central role in cell proliferation control [32, 33]. Constitutively activated AKT is associated strongly with cell proliferation and apoptosis in cancer, and plays a significant role in melanoma[34]. Focal adhesion kinase (FAK) is a key mediator of integrin signaling pathway, which plays a key role in the regulation of cell motility and invasion through the activation of numerous downstream pathways including the ERK and AKT pathways[35, 36]. The role of OVOS2 in these pathways has not been reported previously; however, activated α2-M, to which OVOS2 belongs, can bind to GRP78 and activate signaling cascades including MAPK and AKT-dependent signaling[7–12]. We concluded that the involved mechanisms of OVOS2 affecting proliferation and migration of melanoma cell may at least partly associated with the overactivation of FAK/ERK and FAK/AKT signals. Further investigations are needed to examine whether any interaction exists between OVOS2and GRP78 on the surface of melanoma cells.

The information about our target gene *OVOS2* is very limited, the possible mechanism and binding proteins were totally unknown as well, and thus, the current manuscript is relatively exploratory and has many limitations. For instance, only one type of cell line was chosen for phenotypic studies and other cell lines or primary tumor cells should be used for further investigations. We should reconfirm the results above by performing the OVOS2 over-expression experiment. Inhibitors or activating agents could be used to further study the effects of *OVOS2* on the related signaling pathways. Technologies such as mass spectrometry analysis, co-immunoprecipitation, and molecular docking simulation could also be applied to identify the possible interacting proteins in our future studies.

## Conclusion

In summary, our study revealed that *OVOS2* was overexpressed in melanoma cells, and it could regulate the proliferation and migration of melanoma cells, suggesting a crucial promotive role of *OVOS2* in the progression of melanoma. These results not only represent a new insight and area for molecular researches of melanoma, but also provide valuable theoretical basis and experimental data for further seeking the precise function and molecular mechanisms of the involvement of *OVOS2* on melanoma.

## Supporting information

S1 Fig The representative images of wound healing assay in complete medium (100×). The wounding space between cell layers were almost occupied by the continuous movements of migrated cells after 24 h culture in untreated A375 cell and A375/NC-shRNA cell groups, while the number of migrated cells and the average migration distance were both decreased obviously at 6h, 12h and 24h in A375/OVOS2-shRNA group. Quantitative analysis indicated the migration speed was suppressed significantly in A375/OVOS2-shRNA cells (*P*<0.001, repeated measure of ANOVA).
(TIF)

S1 File Available data. (RAR)
