# Supplementary figures and images for: Ovostatin 2 knockdown significantly inhibits the growth, migration, and tumorigenicity of cutaneous malignant melanoma cells
Source: PLoS One. 2018 Apr 23;13(4):e0195610. doi: 10.1371/journal.pone.0195610 (PMC5912766; doi:10.1371/journal.pone.0195610)

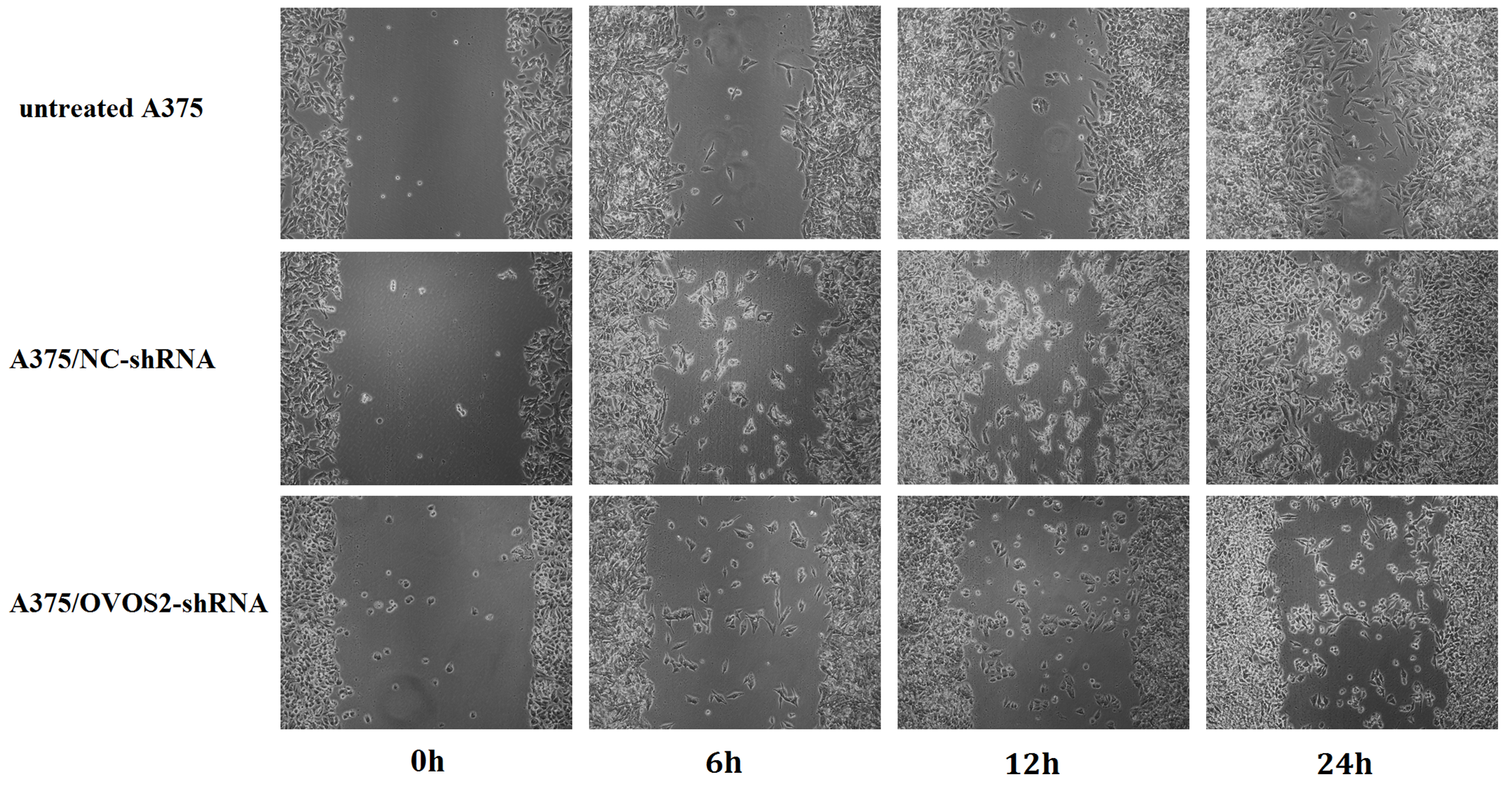

Supplement: S1 Fig — The wounding space between cell layers were almost occupied by the continuous movements of migrated cells after 24 h culture in untreated A375 cell and A375/NC-shRNA cell groups, while the number of migrated cells and the average migration distance were both decreased obviously at 6h, 12h and 24h in A375/OVOS2-shRNA group. Quantitative analysis indicated the migration speed was suppressed significantly in A375/OVOS2-shRNA cells (P<0.001, repeated measure of ANOVA). (TIF) [file pone.0195610.s001.tif]
